# Medical Items

**Published:** 1914-12

**Authors:** 


### MEDICAL ITEMS

### THE REMEDY OF CHOICE IN CARDIAC AFFECTIONS

It is interesting to note the growing interest medical men are taking in Cactina Pillets as a safe and dependable cardiac tonic. This is not surprising; indeed the only surprising feature is that the efficiency of this remedy has not been more generally relized. Hardly ane one drug, with the possible exception of digitalis, has a broader field of activity, and there are many competent observers who place it first among cardiac remedies. Experience has shown that the most conspicuous influence of Cactina upon the heart is its effect on the local nutrition and consequent increase of the muscular-motor energy. Certainly it is the heart tonic par excellence, since it increases heart action and restores nerve function with a promptness that is rarely observed with any other remedy.

Made from a dependable preparation of Mexican Cereus Grandiflorus, Cactina Pillets are especially effective in functional disorders of the heart associated with feeble, irregular pulse, more or less dyspnea and a sense of chest oppression. In such cases the effect of Cactina Pillets is exceedingly gratifying, the heart being promptly steadied and strengthened, and dyspnea markedly relieved. Tachycardia and palpitation are quickly controlled, and the precordial sensations which cause so much apprehension are soon dispelled.

In accomplishing the foregoing, the physician does not have to apprehend toxic or untoward effects, for Cactina Pillets are not only non-cumulative, but totally devoid of all unpleasant or disagreeable action. It is hardly to be wondered at, therefore, that careful, painstaking physicians are not only using Cactina Pillets more extensively than ever, but are gradually coming to look upon this preparation of cactus as the remedy of choice in functional affections of the heart.

---

A great many medical men may be of the opinion that there is very little difference in empty capsules. The prescription clerk knows different and in the best prescription drug stores Lilly Empty Gelatin Capsules are used exclusively because they add to the appearance of prescriptions, are uniform, highly transparent, easy to join and close securely. Physicians who prescribe capsules are favorably impressed with the rapid solubility of the thin walled Lilly capsules and the fact that they are made of the purest gelatin and are clean.

Lilly Empty Capsules are made on automatic machines of the latest improved type, in an atmosphere of washed and sterile air. They are untouched by human hands from start to finish. Eli Lilly & Company will be very glad to send a sample of Lilly capsules upon request addressed to the home office at Indianapolis.

